# Gene Expression analysis associated with salt stress in a reciprocally crossed rice population

**DOI:** 10.1038/s41598-019-44757-4

**Published:** 2019-06-03

**Authors:** Samsad Razzaque, Sabrina M. Elias, Taslima Haque, Sudip Biswas, G. M. Nurnabi Azad Jewel, Sazzadur Rahman, Xiaoyu Weng, Abdelbagi M. Ismail, Harkamal Walia, Thomas E. Juenger, Zeba I. Seraj

**Affiliations:** 1 Plant Biotechnology Lab, Department of Biochemistry and Molecular Biology, University of Dhaka, Dhaka, 1000 Bangladesh; 2 Department of Integrative Biology, The University of Texas at Austin, Austin, Texas 78712 USA; 3 Plant Physiology Division, Bangladesh Rice Research Institute, Gazipur, Bangladesh; 4 International Rice Research Institute, Los Banos, Laguna, Philippines; 5 Department of Agronomy and Horticulture, University of Nebraska, Lincoln, Nebraska 68583 USA

**Keywords:** Natural variation in plants, Salt

## Abstract

The rice landrace Horkuch, endemic to the southern saline coast of Bangladesh, is known to have salt tolerance traits and can therefore contribute to a high yielding recipient for breeding purposes. In this study, we reciprocally crossed Horkuch with high yielding but salt sensitive IR29 to detect the complement of genes that were responsible for conferring salt tolerance versus sensitivity at the seedling developmental stage. We looked at tolerant and sensitive F_3_ families from individual F_2_ segregating plants and analyzed them for differential gene expressions using RNAseq. In general, we observed higher numbers of genes differentially expressed in leaves compared to root tissues. This included both upregulation and downregulation of gene expression across our experimental factors. Gene expression decreased in sensitive leaf after stress exposure where tolerant plants showed the opposite trend. In root, tolerant plants expression decreased at higher time points of stress exposure. We also observed a strong maternal cytoplasmic effect on gene expression and this was most evident in roots where there was upregulation in functional enrichments related to phosphorylation, electron carriers, transporter and cation transmembrane activities. Stress groups (tolerant and sensitive) response in F_3_ families were distinctive in both cytoplasmic backgrounds and involved uniquely upregulated genes in tolerant progenies including membrane sensor proteins, enzymes involved with signaling pathways, such as those producing trehalose and G-protein coupled receptor proteins, photosynthesis-related enzymes and golgi body recycling as well as prolamin precursor proteins involved in refolding of proteins. On the other hand, sensitivity was found to be associated with differential upregulation of only a few redox proteins and higher number of apoptosis related genes compared to the tolerant response. Overall, our highly replicated experimental design was powerful and allowed the detection of relatively subtle differential expression. Our future goal is to correlate these expression differences with QTLs in this population, which would help identify the relative importance of specific genetic loci and provide a direct avenue for combining higher levels of salt tolerance with better agronomic traits in rice.

## Introduction

Nearly 1 million ha of coastal soil in Bangladesh or nearly a ninth of the total cultivable area is affected by soil salinity, mostly due to sea-water intrusion^1^. The inhibitory levels of salinity for rice cultivation (>4 dS/m) are seasonal, except in certain pockets of the South West^2^. During the dry season water levels are usually low due to upper riparian withdrawal as well as its overexploitation, causing a build-up of salts. However, these coastal areas are home to many salt tolerant rice landraces, including both *indica* and aromatic subgroups, which have allelic diversity at several genetic loci associated with tolerance from donor landraces like Pokkali and Nona Bokra^3,4^. One of these rice landraces, Horkuch, was previously characterized as salt tolerant at the seedling stage^3,5^ and at the reproductive stage^6^. These rice landraces from coastal Bangladesh are likely to harbor novel sources of salt tolerance, due to their allelic diversity, that can complement known salt tolerance genes. The introduction of new sources of salt tolerance to breeding programs for rice are essential to ensure food security not only for the increasing population, but also the steadily rising levels of salinity inwards from the Bangladesh coast^7^.

Rice growth is the most sensitive to salinity stress at two developmental stages, early seedling and during reproduction^8–10^. However, seedling and reproductive stage tolerance are poorly correlated because separate sets of genes may be involved at different developmental stages in coping with salt stress^11^. It is therefore important to identify both seedling and reproductive stress tolerance traits and combine them in breeding lines for durable tolerance. Seedling stage salt tolerance has been studied extensively in rice, mainly in the common donors, like Pokkali and Nona Bokra^11,12^. The physiological response to salt stress is complex, with an immediate osmotic stress, manifested by reduced water uptake, lowering of cell expansion and growth retardation^13^. Tolerant cultivars likely respond to this first phase of salt stress by controlling their stomatal apertures and producing compatible solutes^13,14^. Plants subsequently experience ionic stress due to a gradual buildup of Na^+^ over a period of days and weeks. A common mechanism for salt tolerance in rice is maintenance of lower shoot Na^+^ content^15,16^, which can be due to sodium exclusion^17^, effective sequestration of toxic salts into older leaves^18,19^ and roots^20^ and compartmentalization and extrusion of Na^+^ into vacuoles and out of cells^21^. Despite these common physiological trends in response to salt stress in tolerant rice cultivars, there is wide variability in injury scores and the amount of Na^+^ in the seedling, particularly the 3^rd^ leaf referred to as 3leafNa^22^. In general, rice with higher biomass were shown to have lower 3leafNa, but those with highly variable salt injury scores (SES), had a wide range of low to high 3leafNa concentrations^22^. Therefore, there is a case to be made for independent study of many tolerant genotypes, which may have novel mechanism for combatting salt stress.

In addition to its toxicity, sodium itself has been proposed to function as a signaling molecule of salt stress after perception by non-selective cation channels (NSCC) of the depolarization type, which acts in a few seconds in the membrane^23,24^. This signaling included activation of mechanosensitive Ca^2+^ transporters, Ca^2+^ influx, activation of the SOS pathway, upregulation of NHX1 and the MAPK pathway, production of ABA and NADPH oxidase and generation of ROS, each having several downstream effects in tolerant grape^24^. In rice, salt tolerance was reported to be associated with high expression of CYP94C2b and concomitant deactivation of jasmonate^25^. It is also known that the tolerance or sensitivity of a rice genotype is dependent on the complex signaling and downstream responses of a number of different systems involving Ca^2+^-binding proteins^26^, ABA^25^, ROS species and their scavengers^27,28^, various transporters^26^, protection and preservation of the photosynthesis apparatus^29^ as well as management of energy production^26^. Despite progress in understanding the molecular signaling and mechanisms of salt tolerance, much remains to be learned from the natural and adaptive salt tolerance observed in landraces.

The rice landrace, Horkuch was grouped with aromatic rice and hence is divergent from the common *indica* salt tolerance donors such as Pokkali and Nona Bokra used in breeding lines developed at IRRI and in Bangladesh^16^. As Horkuch exhibits salt tolerance at both seedling^3,19^ and reproductive stages^6^, it will be useful to identify the salt tolerance determinants of Horkuch for use in breeding programs. As established above, all evidence suggest that salt tolerance is not only polygenic and impacted by multiple independent physiological mechanisms but is also highly variable among genotypes. A long-term goal would be to disentangle the causal factors driving this variation within known salt tolerant land races. An ideal strategy for accomplishing these goals would be to recombine genetic loci controlling this variation in genetic mapping populations.

In this study, we have used reciprocally-crossed recombinant progenies between tolerant Horkuch and sensitive, but high-yielding IR29. F_3_ families were chosen to represent 15 segregating F_2_ individuals for both the tolerant and sensitive seedling category based on their injury scores (SES) at F_2_ and used for RNAseq analysis following a novel 3′ tag-seq method^30^. Our aim was to identify genes involved in response to salinity stress in the leaf and root tissues resulting in tolerance versus sensitive response. We identified significant differential salinity stress responses associated with sensitive and tolerant genotypes in both reciprocal crosses. Overall, uniquely upregulated genes in tolerant progenies included membrane sensor proteins, enzymes involved with signaling pathways, such as those producing trehalose and G-protein coupled receptor proteins, photosynthesis-related enzymes and golgi-body recycling as well as prolamin precursor proteins involved in re-folding of proteins. On the other hand, sensitivity was found to be associated with differential upregulation of lower number of redox proteins and higher number of apoptosis-related genes compared to the tolerance response. Generally, our findings will help identify the relative importance of specific genes which can help genotypes combine higher salt tolerance with the best agronomic traits.

## Materials and Methods

### Development of the reciprocally crossed population

We chose a salt tolerant Bangladeshi rice landrace and a high yielding but salt sensitive variety developed by IRRI to generate a reciprocally crossed population for this study. The salt tolerant parent is Horkuch^3^ which is also known as Horkocha^5^ or Horcoach^4^. In this study, Horkuch (IRGC 31804) and IR29 (IRGC 30412) were collected from the IRRI Gene bank for hybridization. The accession of Horkuch used in this work was independently genotyped using the 384 SNP rice platform and found to cluster closer to the Japonica subgroup, consistent with the work of Rahman *et al*.^16^ (Adam Price, 2013, personal communication). Seeds were sown in a crossing block after breaking dormancy at 50 °C for 5 days in an oven during the wet season of 2011 (June-July) at IRRI, in the Philippines. F_1_ plants were initially confirmed by SSR marker (RM493) and both F_1_s were advanced to the F_2_ stage by selfing. 1200 F_2_ progenies from both crosses (600 from each cross direction) were advanced to F_3_ at BRRI in Bangladesh. Then a phenotypic screening was conducted with a subset (300 randomly selected F_3_ families) under salt (NaCl) stress to determine tolerant and sensitive F_3_ progenies at the seedling stage. Henceforth, RNAseq data of F_3_ progenies derived from Horkuch × IR29 are referred to as Horkuch♀ and those from the reciprocal cross as IR29♀.

### Initial physiological screening and selection of plants for expression study

Plants were grown in hydroponic solution using 3 siblings as replicates of each family. Two weeks after germination (at four leaf stage), 12 dS/m salt stress (NaCl) was applied gradually in 2 dS/m increment per day, starting from 6 dS. After 2 weeks of salt stress, plants were scored using the SES (Standard Evaluation System) protocol^31^ which evaluates leaf damage of plants. Here a scale of 1–9 corresponds to ascending salt sensitivity. To make the SES score clearer, a reverse SES score was compiled and termed as tolerance score. From these initial screening results, 60 F_3_ families were selected, 30 from each cross direction population based on the SES values. This selection contained tolerant and sensitive families from the two extreme tails of the SES score distribution (Supplementary Fig. 1). Physiological and morphological parameters like stomatal conductance, chlorophyll content, sodium and potassium content, shoot and root relative water content, dry weight, shoot and root length of the salt stressed plants were measured for the selected progenies.

Relative water content (RWC) of shoot and root were calculated using the sample fresh weight (W), the turgid weight after 24 hrs hydration (TW) and the oven dried weight at 80 °C for 24 hrs (DW). The formula adopted was RWC = [(W − DW)/(TW − DW)] × 100, where, W = sample fresh weight, TW = Sample turgid weight, DW = Sample dry weight.

Stomatal conductance of fully opened young leaves were measured after 4 days of salt stress by a Decagon Leaf Porometer (sensor serial LPS1283) (Decagon Inc. USA) on a bright sunny day from 11 am to 2 pm. Chlorophyll content was measured by cutting one leaf into ~1 cm^2^ pieces and weighing followed by soaking in 20 mL 80% acetone in dark. After 48 hrs, absorbance was taken at 645 nm, 663 nm for chlorophyll a and b and 652 nm for total chlorophyll^32,33^.

Plants were washed in flowing tap water followed by oven drying for the measurement of sodium and potassium concentrations in seedling stage leaf. Dried leaves from each replicate were pooled, ground and analyzed by a flame photometer (Sherwood model 410, Sherwood, UK) after 48 hrs of extraction with 1 N HCl. Concentrations of Na^+^ and K^+^ were expressed as mmol/g dry weight. Leaf and root lengths were measured after harvesting the plants and dry weights were measured after drying the shoots and roots completely in an oven.

### Experimental designs and RNA isolation

**Figure 1:**
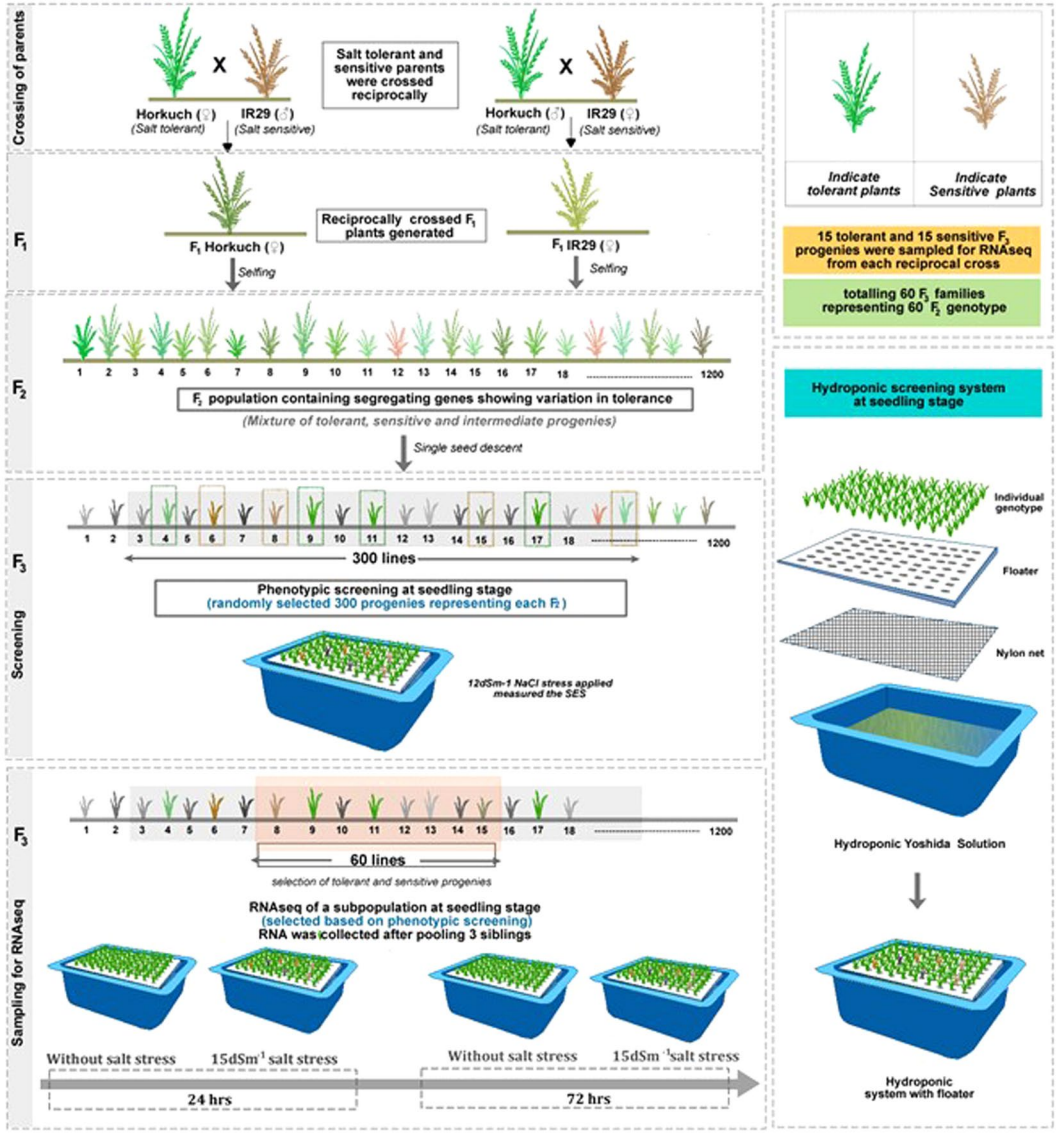
A depiction of the overall experimental design from crossing to the sample collection strategies. In the F_2_ reciprocal populations, tolerant and sensitive plants were categorized by SES value. The experimental design has been shown sequentially from crossing to F_3_ progenies in separate sections.

Our gene expression studies focus on 15 tolerant and 15 sensitive F_3_ families from each reciprocal cross, identified in our initial stress tolerance screen. The experimental design is shown in Fig. 1 and further explained with the sample sizes of progenies from each category in Supplementary Fig. 2. The experiment included a number of experimental factors including cytoplasm (cross direction), treatment (control or salinity stress), time points (24 hrs or 72 hrs) and stress group (sensitive or tolerant).

Three siblings from each of the selected F_3_ families were grown in hydroponics in Yoshida culture solution^37^ in a net house at the University of Dhaka, Bangladesh. These progenies were planted randomly following an incomplete block design to avoid possible environmental bias. We grew two sets of the progenies, one was for the salinity treatment and the other one was for collecting control samples. Temperature and humidity recorded at that period averaged 27 °C at night and 34.9 °C during the day. After 15 days of germination, 15 dS/m of salt stress (NaCl) was applied gradually, in 5 dS/m increments per day to individual plants. Samples (replicated pools of 3) were collected at two time points, at 24 hrs and 72 hrs after applying the targeted salt (NaCl) stress. Control samples or samples without salinity treatment were also collected at both time points. Shoot and root tissues were collected separately. We collected the whole shoot and root materials and extracted RNA for sequencing. Therefore, the total number of samples were [2 stress groups (tolerant/sensitive) × 2 cross directions × 2 treatments × 2 time points × 15 biological replicates corresponding to F_3_ families = 240 samples × 2 tissues = 480. The number of samples from each experimental design has been shown in Supplementary Fig. 2 for further clarification. RNA was isolated from the collected tissues using TRIZOL following manufacturer’s protocol (Invitrogen, USA) and quantified using Nanodrop® spectrophotometer ND‐1000 (Thermo Fisher Scientific Inc.). Isolated RNAs were shipped to University of Texas at Austin (UT, Austin) using RNAstable^®^ following the manufacturer’s protocol (Biomatrica, USA) to initiate library preparation for sequencing. After receipt of samples at UT Austin, RNAs were treated with DNAse I (Promega, USA) at a concentration of 1 unit/μg of total RNA. Total RNA purity and degradation were again evaluated on 1% agarose gels before proceeding to library preparation.

### Library preparation and sequencing with Illumina Hiseq-2500 platform

For each sample, 1 µg of total RNA was used for RNA-Seq library preparation following the protocol described by Meyer *et al*.^34^. According to the protocol, RNAs were first heat fragmented. Then, first strand cDNAs were synthesized using a modified oligo-dT containing a primer targeting 3′ ends. Prepared cDNAs were later amplified with sample specific oligonucleotide barcodes, then quantified and pooled prior to sequencing. In this study, 32 barcodes from Illumina were used for library preparation purposes. For a single lane, 32 samples were pooled for sequencing by Illumina next-generation sequencing platform (Hiseq) as 1 × 100 bp reads at the University of Texas, Austin’s Genome Sequencing and Analysis Facility (GSAF) service. Each lane (16 lanes total) of ~32 samples generated an average of ~190 million raw reads from both leaf and root samples.

### Quality check, filtering, mapping and transcript counts

Raw sequencing reads were initially screened by FastQC^35^. Filtering involved several criteria including removing reads with homopolymer counts >20% and Poly A counts >20%. In addition, reads with sequence quality (>20) and <5 missing values were retained. The minimum length after filtering was set at >30 bp. Here it is noted that before deciding the length after filtering, K-mer profiling was completed with available rice transcriptomes to decide the minimum length cutoff and it was revealed that about 90% of rice transcripts are unique at 30 bp length^36^. Filtered libraries were mapped against the genome of Nipponbare/*japonica* subspecies of *Oryza sativa* using bowtie/1.0.0^37^ short read aligner. Uniquely mapped reads were filtered by samtools flag ‘bq’ at 10^38^. The rice annotation file (*Oryza sativa)* from Phytozome V.9^39^ was used to generate count files from sequenced RNA.

### Distribution model, DEGs in different interactions

After generating count files from the RNAseq reads, the data were subsequently culled to remove transcripts with low counts (sum of counts less than 100 across samples). Filtered samples were KDMM normalized using JMP Genomics 7.0 (SAS, Cary NC) and further filtered by removing transcripts with 30% zero counts. This left ~11,000 transcripts for leaf samples and ~7700 transcripts for root samples. A linear mixed model was fit for each transcript using a negative binomial distribution^40^ and a log link function. The model included factors for cytoplasm (IR29 versus Horkuch), treatment (salt stress versus control), time point (24 hrs versus 72 hrs), stress group (sensitive versus tolerant) and all possible pairwise and a 3-way interactions along with a random effect for sequencing lane. The basic model was as constructed as: expression = constant + stress group + treatment + cross-direction + time + stress group*treatment + stress group*cross-direction + stress group*time + treatment*cross-direction + treatment*time + cross-direction*time + stress group*time*treatment + lane + error. Here, lane and error are considered random effects. The remaining three-way or higher interactions were not studied and so are pooled with the residual. Differentially expressed genes from the above factors were identified using a false-discovery rate (FDR) of 0.05. In addition, we chose some specific contrast groups from the two and three ways interactions of main effects. These selected groups helped us decouple stress group and cross direction effects. For example, we chose to contrast Horkuch♀ stress vs control and IR29♀ stress vs control from the cross-direction*treatment interactions. We highlight the contrast groups in our results section and discussed their differentially expressed genes.

### GO annotation and enrichment analysis

Differentially expressed genes from main effects and their interactions were studied for gene-set enrichments by AgriGO^41^ using a hypergeometric test after Hochberg FDR correction with a significance level of p < 0.05. GO enrichment sets were further summarized using ReviGO^42^ by removing redundant GO terms. For ReviGO analysis, *Oryza sativa* database was selected as GO term size using SimRel^43^ as a standard for semantic similarity measurement.

## Results

### Physiological responses and seedling scoring for salinity tolerance

**Figure 2:**
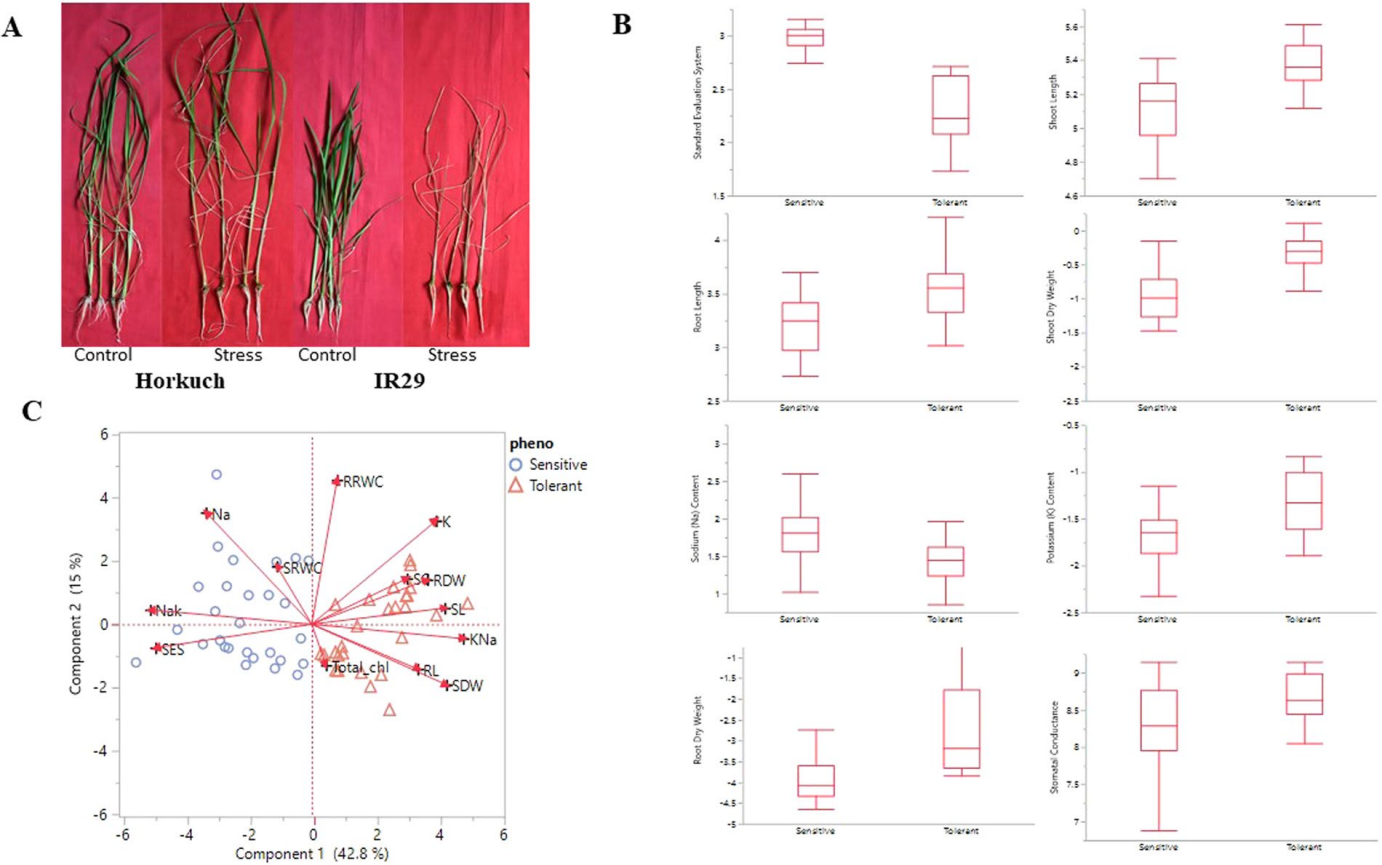
Phenotypic data variability and categorization of segregating populations under salinity stress at seedling stage level. Fig. **A** Shows the performances of the parental lines of the reciprocal cross populations under salinity and control condition. Horkuch performed similarly to its unstressed control but IR29 showed strong sensitivity to the salinity stress. Fig. **B** Plots various phenotypes that differ between sensitive and tolerant progenies at a p-value of less than 0.05. Fig. **C** PCA plot generated with all phenotypes at the seedling stage under salinity stress condition. PC1 explains 42.8% of variability of the data and clearly partitions sensitive and tolerant progenies.

The parental lines show a strikingly different degree of tolerance under salinity stress at the seedling stage. Horkuch does not exhibit significant phenotypic responses to high salinity stress in contrast to the sensitive parent (IR29), which does not survive the imposed salinity stress (Fig. 2A) at the stress levels studied. In the reciprocal F_2_ populations, we categorized tolerant and sensitive plants from both cross directions in F_3_ families (each family representing an F_2_ individual) under salinity stress at the seedling stage by evaluating SES (Standard Evaluation System) value following IRRI protocol (details in Method section). We collected data for 13 physiological and morphological traits from the F_3_ reciprocal families at the seedling stage under salinity stress. We found that 8 out of 13 traits were significantly different (p-value < 0.05) between tolerant and sensitive progenies (Fig. 2B). Principal component (PC) analysis of these phenotypic traits showed strong variability between tolerant and sensitive progenies. The first two PC explain 57% of data and PC1 (which explains 42.8% of the variation) clearly partitions tolerant and sensitive lines in two different categories (Fig. 2C). In PC1, two major clusters were observed where SES value clustered closely with Na^+^ (sodium) and Na/K (sodium/potassium) ratios. Overall, our phenotypic data suggests that lower SES values and higher values of traits like SC (Stomatal Conductance), RDW (Root Dry Weight) and SL (Shoot Length) combine tolerant characters (Fig. 2) (Phenotypic data are at Supplementary File 1).

An ascending trend with increased level of tolerance was observed for dry weights and shoot and root length, potassium content and stomatal conductance. High salinity levels caused simultaneous reduction in seedling root and shoot dry biomass production. Relative water content (RWC), the measure of water status in terms of the cellular hydration as a consequence of leaf water potential and osmotic adjustment, normally decreases at higher salinity levels^11^. However, there was no trend of either SRWC or RRWC with increasing tolerance in our experiments. Moreover, no clear distinction between tolerant and sensitive plants with respect to RWC was observed. Total chlorophyll also did not show a trend with increased tolerance values (Fig. 2) (Phenotypic data are at Supplementary File 1). This indicated that chlorophyll and water content are not sufficient to confer salt tolerance to this rice genotype at least for the time points considered in this study.

### General patterns of transcripts count in different tissue types and their correlations

**Figure 3:**
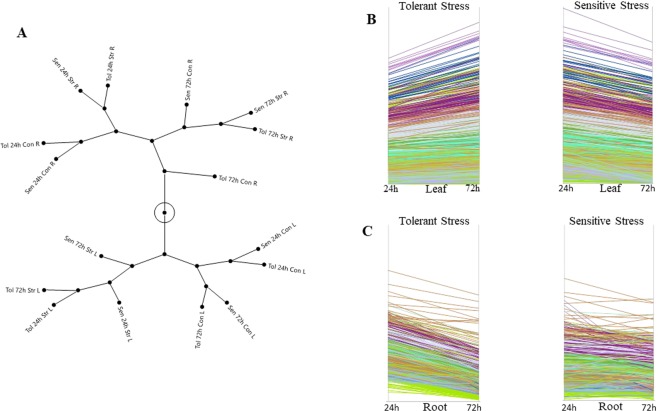
Multivariate structure of transcripts counts from both leaf and root tissue across experimental factors. Fig. **A** Presents the expression cluster analysis as a constellation plot among all experimental groups. Leaf and root tissues clustered very differently. In leaf tissue, the salt stress treatment partitioned the experimental groups while in root tissue sampling time differentiated experimental groups. Fig. **B** & **C** Show the overall trend of expression variation in sensitive and tolerant plants in leaf and root tissues separately. Overall gene expression decreased in sensitive leaf after stress exposure where tolerant plants showed the opposites trend. In root, tolerant plants expression decreased at higher time points of stress exposure.

We calculated simple Pearson correlations among experimental factors by pairwise comparison. Leaf and root samples were poorly correlated compared to those within the same tissue type. The correlation coefficients were greater than 0.94 and 0.90 across all transcripts in leaf and root tissues respectively. In contrast, leaf and root had correlation coefficients in the range of 0.45–0.61 (Supplementary Fig. 3). We also clustered the experimental groups to gain insight on the multivariate data structure. Different clusters were represented as a constellation plot (Fig. 3A) revealing that leaf and root tissue experimental factors were partitioned as two major groups. In leaf tissue, controlled and stressed samples are clustered separately. In control conditions, sensitive 24 hrs and 72 hrs samples were grouped with tolerant 24 hrs and 72 hrs samples respectively, which indicates that sensitive and tolerant plants were behaving similarly when no stress was applied. In the stress condition, tolerant and sensitive plants were clustered separately indicating that sensitive plants under stress had variable expression patterns in different time points. In root tissue, experimental groups were clustered based on time points. 24 hrs and 72 hrs samples were clustered separately. At 24 hrs, no obvious patterns between sensitive and tolerant plants under different treatment conditions were observed. However, at 72 hrs, sensitive and tolerant samples responded differently to the stress condition (Fig. 3B). Given the relatively unique response patterns of leaf and root tissue, we subsequently analyzed leaf and root tissues separately.

To initially explore the global patterns of gene expression among tolerant and sensitive progenies, we selected the top 3,000 highly expressed transcripts in both leaf and root tissues to see the patterns of different stress groups under stress. In leaf tissue, sensitive and tolerant progenies showed opposite expression patterns at 24 hrs compared to 72 hrs. Sensitive plants generally had lower expression counts after 72 hrs of stress compared to 24 hrs but tolerant plants had higher expression counts at 72 hrs of stress rather than at 24 hrs. The gradual increase in gene expression over time observed in leaf tissue of tolerant plants is absent in sensitive plants. In root tissue, sensitive plants had mixed expression responses at the two time points (24 hrs and 72 hrs) while tolerant plants had a very clear decrease of gene expression at 72 hrs of stress (Fig. 3C).

### Patterns of differentially expressed genes in main effects

**Figure 4:**
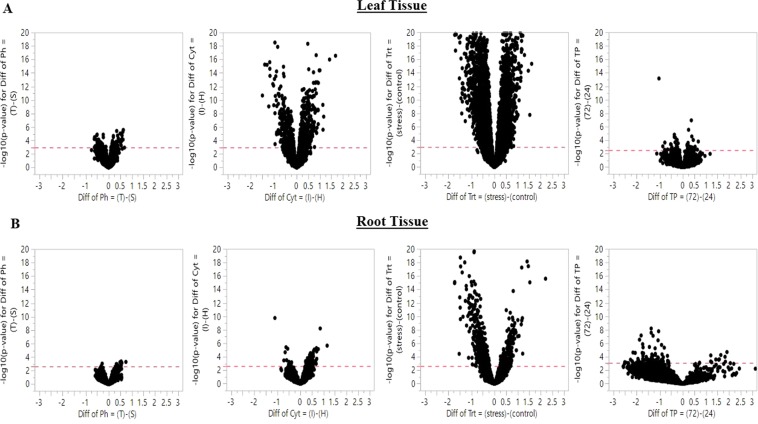
Volcano plots of significant genes in leaf (Fig. **A**) and root tissue (Fig. **B**) from the main experimental factors. The x-axis represents fold change (Fc) and the y-axis represents negative log10 of the P-value of each gene. In general, main effects showed stronger signals in leaf tissue compared to root. However, time point differences in leaf had fewer differential expressed genes (**first from the top right**) compared to root (**first from the bottom right**).

Differentially expressed genes (DEGs) were identified from main effects (factors of the experimental design) and contrast group chosen from their pairwise interactions. Significant DEGs were observed for all model factors (Table 1, Fig. 4) and in both tissue types, with an especially strong DEG signal in leaf tissue. The salinity treatment exhibited the largest number of DEGs for both leaf and root tissue, while time point showed greater DEGs in root tissues compared to leaf (Fig. 4). Under salinity treatment, more genes were variably expressed in leaf compared to root tissue; 3687 genes were differentially expressed in leaf and 695 genes in root. Interestingly, more genes were downregulated in the stress condition compared to their control counterpart in leaf and root tissue. In leaf 56% of the DEGs were downregulated in stress condition while 72% of DEGs were downregulated in root tissue. For the reciprocal cross direction effect, 777 genes were differentially expressed in leaf tissue and 91 genes in root tissue. The number of upregulated genes was higher from IR29♀ cross in leaf and root tissue compared to Horkuch♀ cross. In leaf tissues, 57% of the differentially expressed genes were upregulated in IR29♀ compared to Horkuch♀ progenies whereas in root tissue this was 82% (Table 1).

**Table 1:** Number of differentially expressed genes in leaf and root tissues based on FDR cutoff >0.05 for various design factors.

Factor	Comparison	DEGs (n) in Leaf Samples (down/up regulated)	DEGs (n) in Root samples (down/up regulated)
Cross-direction	IR29 (IR) ♀vs Horkuch (H)♀	↓329 ↑448	↓16 ↑75
Treatment	Stress (St) vs Control (Cn)	↓2091 ↑1596	↓505 ↑190
Stress group	Tolerant (Tol) vs Sensitive (Sen)	↓36 ↑41	↓2 ↑9
Time Point	72 hours vs 24 hours	↓13 ↑14	↓144 ↑114
Cross-direction × Treatment	Horkuch♀ Stress vs Horkuch♀ Control	↓1856 ↑1598	↓357 ↑58
	IR29♀ Stress vs IR29♀ Control	↓2005 ↑1574	↓119 ↑52
Stress group × Treatment	Sensitive Stress vs Sensitive Control	↓1899 ↑1574	↓225 ↑46
	Tolerant Stress vs Tolerant Control	↓1890 ↑1509	↓196 ↑74
Treatment × Time Point	Control 24 vs control 72	↓47 ↑53	↓105 ↑36
	Stress 24 vs Stress 72	↓88 ↑105	↓167 ↑34
Stress group × Time Point	Sensitive 24 vs Sensitive 72	↓21 ↑18	↓32 ↑9
	Tolerant 24 vs Tolerant 72	↓13 ↑10	↓99 ↑43
Stress group × Time × Treatment	Tol 24hrs St vs Sen 24hrs St	↓30 ↑23	↓0 ↑4
	Tol 72hrs St vs Sen 72hrs St	↓18↑10	↓2 ↑16

Downregulated and upregulated genes are indicated with directional arrows.

The other two main effects, stress group and time point, detected fewer DEGs compared to cross direction and treatment effect. Between stress groups, 77 genes were differentially expressed in leaf and only 11 genes in root. It was notable that the effect of time points on roots showed stronger signals than in leaf tissue. Only 27 genes were differentially expressed in leaf where 14 genes were upregulated and 13 genes were downregulated in 72 hrs compared to the 24 hrs counterpart. In root 258 genes were differentially expressed where 114 genes were upregulated in 72 hrs and 144 genes were upregulated in 24 hrs. The total numbers of DEGs observed in leaf tissue is 10% of total DEGs observed in root tissue under time differences (Table 1). Thus time point among all other main effects showed higher degree of differential genes in root tissue compared to that of leaf.

### Effect of stress group as a main effect in leaf and root tissue

A major focus of our study was to identify genes associated with tolerance and sensitive plants and their response to salt stress as these are likely candidates underlying the remarkable salt tolerance of Horkuch. The number of genes strictly differing between stress groups were comparatively lower than other main effects like treatment and cross-direction. Therefore, we carefully inspected DEGs in sensitive and tolerant progenies separately in leaf and root tissues.

**Figure 5:**
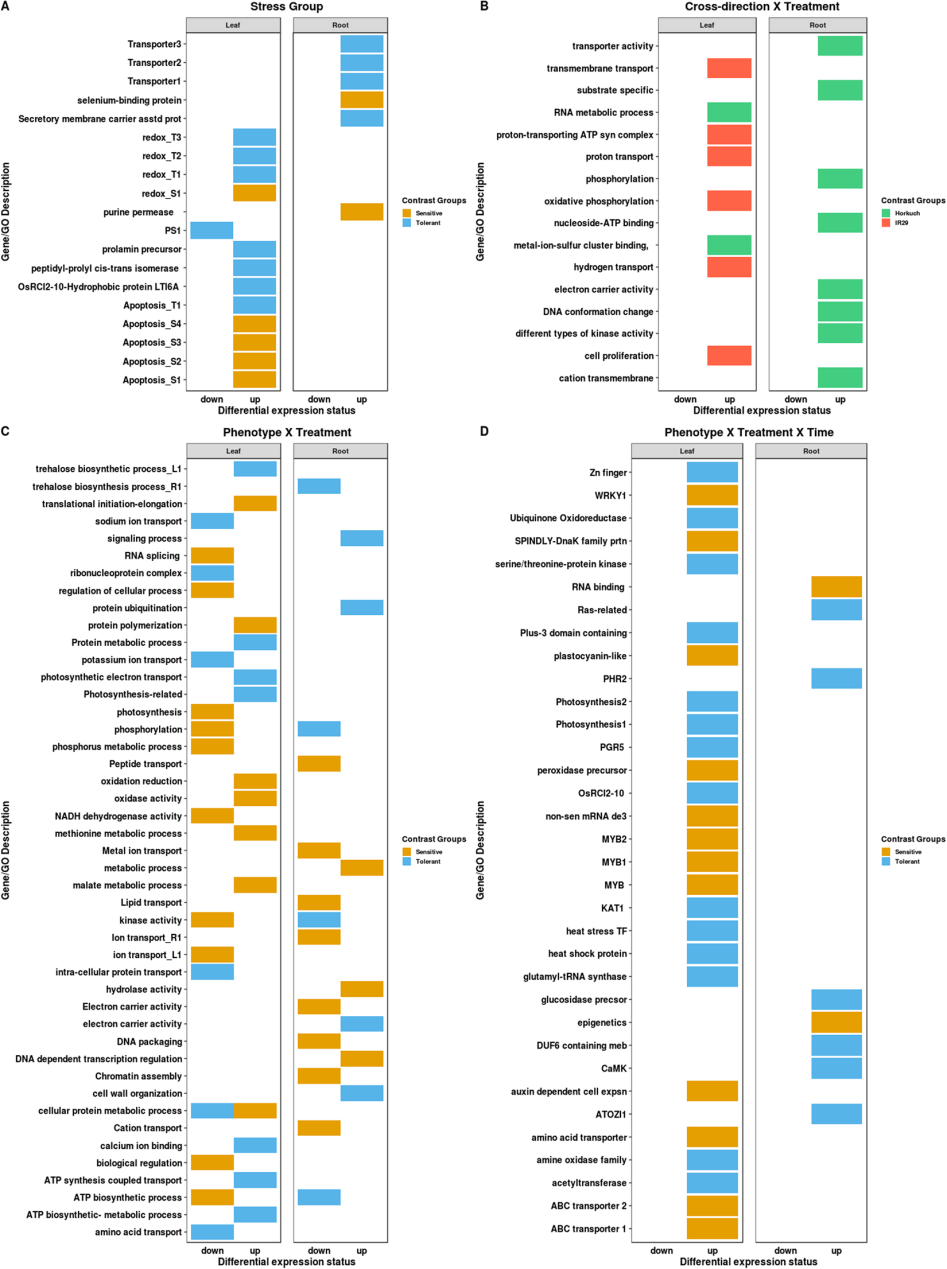
A graphical representation of GO and important genes enrichments in main effects and contrasts from interacting factors. In fig. **A**–**D,** DEGs from stress group, cross-direction × treatment, stress group × treatment and stress group × treatment × time were shown as GO enrichment and gene annotation. GO/gene enrichment in contrasting stress groups is in fig. **A**. Fig. **B** Shows the enrichments of GO and genes for the cross-direction × treatment effect. Treatment × stress group interaction extracted GO/genes were plotted in fig. **C**. In the last part of the fig. **D** shows the differentially expressed genes and their associated GOs revealed from the composite experimental factor (stress group × treatment × time).

In leaf tissue, 77 genes were differentially expressed between tolerant and sensitive plants. 36 genes were upregulated in sensitive plants and 41 genes were upregulated in tolerant plants (Table 1). From the gene annotation file, we found two genes responding to abiotic stress in sensitive plants were upregulated while in tolerant plants there were 6 upregulated genes responding to abiotic stress. There were 4 kinase genes showing upregulation in sensitive plants whereas the number was 2 in tolerant plants. Two transporter activity-related genes were upregulated in sensitive and three were in tolerant plants. A membrane potential associated gene, which is a hydrophobic protein (OsRCI2-10-Hydrophobic protein LTI6A), was upregulated in tolerant leaf tissues. In tolerant plants, 2 photosynthesis related genes were upregulated whereas there were no upregulated photosynthesis genes in sensitive plants. Apoptosis and cell death related genes were upregulated more in sensitive plants (4 genes) compared to tolerant plants (1 gene only). Three oxidation reduction related genes were upregulated in tolerant plants and only one in sensitive plants. We also detected a Prolamin precursor (PROLM23) gene to be only upregulated in tolerant plants (Fig. 5A). We also detected the upregulation of 2 peptidyl-prolyl cis-trans isomerase in tolerant plants. This gene is responsible for accelerating protein folding by catalyzing the cis-trans isomerization of peptide bonds with the amino acid proline (Supplementary File 2).

In root tissue, 11 genes were differentially expressed between sensitive and tolerant plants. 2 genes were upregulated in sensitive plants whereas 9 genes were upregulated in tolerant plants (Table 1). In sensitive plants 2 of the upregulated genes are purine permease (LOC_Os01g48800.1) and selenium-binding protein (LOC_Os01g68770.1) (Fig. 5A). Purine permease has transporter activity and selenium-binding protein responds to stress. Three of the upregulated genes in tolerant plants are associated with transporter activity. Secretory carrier-associated membrane protein (LOC_Os07g37740.1) is also upregulated in tolerant roots and it functions as a carrier in post-golgi recycling pathways to the cell surface (Supplementary File 2).

### Patterns of differentially expressed genes in selected contrasts between main effects

From our linear model, we looked at a set of targeted two-way and a three way interactions between the main effects of the study. We carefully chose interesting contrast groups from the interactions that help decouple stress tolerance behavior between sensitive and tolerant progenies. For example, we inspected genes that were differentially expressed between control and salinity stress conditions in Horkuch♀ and IR29♀ progenies separately. Both Horkuch♀ and IR29♀ showed similar number of differentially expressed genes in leaf tissues with variable expression patterns. Horkuch♀ and IR29♀ progenies exhibited 3454 and 3579 differentially expressed genes in leaf tissue under stress treatment, respectively. In contrast, stronger signals were associated with the stress treatment in Horkuch♀ when compared to IR29♀. There were 415 genes differentially expressed from Horkuch♀ and 171 genes from IR29♀. This indicates a very clear cytoplasmic background bias over expression variation in response to the salinity treatment. In addition, stress group*treatment interaction explained the variable expression patterns between sensitive and tolerant progenies under salinity treatment condition. This is despite the fact that we observed that sensitive and tolerant plants also showed expression of nearly an equal number of genes under stress in leaf and root tissues. In leaf tissues, sensitive progenies had 3473 and tolerant progenies had 3399 DEGs. In root tissue, sensitive progenies had 271 DEGs and tolerant progenies showed 270 DEGs (Table 1).

We also tested for DEGs from the treatment*timepoint and stress group*timepoint interactions perspective. For example, we contrasted control 24 hrs vs control 72 hrs and stress 24 hrs vs stress 72 hrs. This interaction identified DEGs mainly affected by development and stress. We found 100 genes differentially expressed in control and 193 genes in stress conditions between time point differences (24 hrs vs 72 hrs) in leaf tissues. In root tissue, we observed 141 DEGs in control and 201 DEGs stress condition. In addition, stress group*timepoint interaction identified genes that were differentially expressed between sensitive and tolerant plants at different time points. We detected only 39 DEGs in sensitive progenies and 23 DEGs in tolerant progenies when comparing expression profiles at 24 hrs vs 72 hrs in leaf tissue. In root, sensitive progenies had 41 DEGs and tolerant had 142 DEGs contrasting genes at 24 hrs and 72 hrs. Interestingly in root tissues, tolerant plants had a higher number of DEGs compared to sensitive plants when associated with differences in time of stress (Table 1).

From contrasts involving two-way interactions, roots behaved differently from leaf tissues. For example, we detected strong downregulation signals in root tissues for Horkuch♀ and IR29♀ progenies under stress. This pattern also persisted when we compared sensitive and tolerant plants under stress from stress group*treatment interactions. We detected 86% and 69% of DEGs were downregulated in root tissues in Horkuch♀ and IR29♀ populations respectively, while in leaf tissue the percentage of downregulation was 53% and 56% of the total DEGs. Stressed root tissues in sensitive and tolerant plants showed downregulation of DEGs respectively by 83% and 72%, whereas in leaves the downregulation was 54% and 55%, respectively.

### Effect of Cross direction on differential gene expression

**Figure 6:**
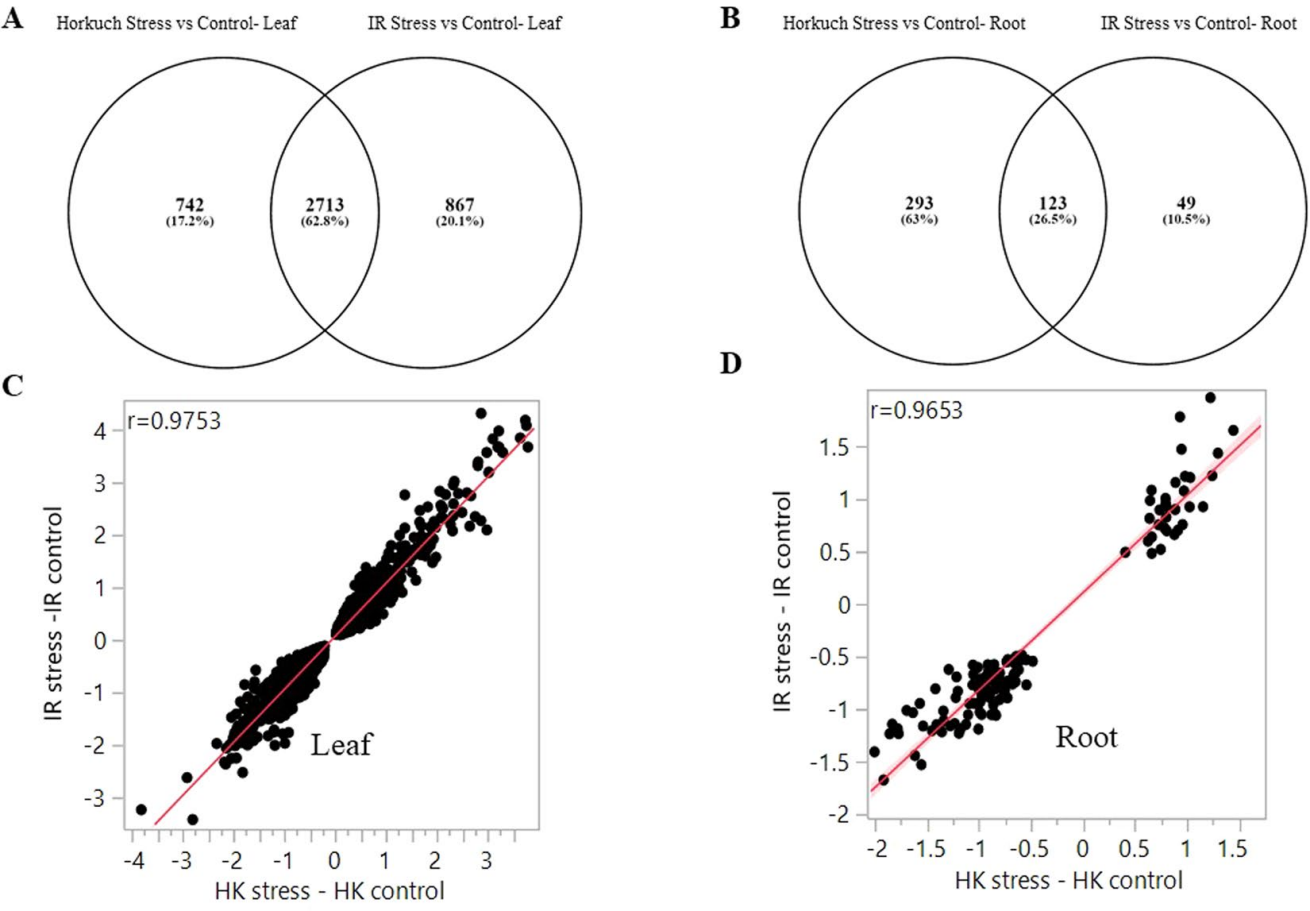
Expression variation influenced by cytoplasmic background and common and unique differential gene counts. Fig. **A** & **B** Shows the expression variations in leaf and root tissues when they got compared by their cytoplasmic background. Similarities and differences of DEGs from two different cross directions plotted here in leaf and root separately. 62% of leaf DEGs were found common in leaf where only 26% were found common in root tissues. Fig. **C** & **D** Fold differences of the common genes from two cross directions were plotted here. In leaf tissue, correlation coefficients were 0.97 and in root it was 0.96.

Our study population came from contrasting cross directions (reciprocal crosses) differing in plastid genomes (both chloroplast and mitochondria) as well as cytoplasmic environments. We studied differential gene counts from the two-way interaction between cross-direction and treatment to obtain a global picture of gene expression variation and functional enrichments influenced by differences in cytoplasmic inheritance, as well as how the effect of the salinity treatment is modified by the cytoplasmic background. For example, we studied differential genes detected from Horkuch♀ stress vs Horkuch♀ control and IR29♀ stress vs IR29♀ control in leaf and root tissues. We observed a very strong effect of cross-direction on gene expression in both leaf and root tissues. In leaf tissues 62.8% genes (Fig. 6A) overlapped between cytoplasmic backgrounds, whereas in root tissue the overlap was only 26.5% (Fig. 6B). These overlapping genes across different backgrounds were found to be highly correlated, in leaf tissue the correlation coefficients were 0.97 (r = 0.9753) and in root tissue it was 0.96 (r = 0.9653) (Fig. 6C,D). Because of these high correlations among the common genes, we also looked for functional enrichments for the genes that were uniquely differentially expressed by cytoplasmic background by gene ontology (GO) study. In leaf tissues, for Horkuch♀, 742 unique genes contributed to 27 significant GO terms and 867 unique genes contributed to 77 significant GO terms for IR29♀ (p-value < 0.05 and FDR < 0.05) (Supplementary File 3). From this GO annotation study, we observed different enrichments from both Horkuch♀ and IR29♀ progenies. In Horkuch♀ leaf tissue, unique GO terms were gene expression, cellular component biogenesis-assembly, transport, metal-ion-sulfur cluster binding, and RNA metabolic process. In IR29♀ leaf tissue, small molecule metabolic processes like cellular ketone, organic acid, nucleobase, nucleoside, nucleotide, cellular amino acid and derivative metabolic process were found enriched (Fig. 5B). There was also hydrogen transport, transmembrane transport, proton transport, cell proliferation, oxidative phosphorylation and proton-transporting ATP synthase complex GO terms enrichments in the unique DEGs of leaf IR29♀ (Supplementary File 3).

In root tissue, Horkuch♀ exhibited 293 unique DEGs where IR29♀ had only 49 DEGs. Unique genes from Horkuch♀ contributed to 37 significant (p-value < 0.05 and FDR < 0.05) GO terms. Interestingly, there were no significant GO terms observed for the unique DEGs from IR29♀. Horkuch♀ had functional enrichments in DNA conformation change, phosphorylation, different types of kinase activity, transporter activity including substrate specific and cation transmembrane, electron carrier activity and nucleoside-ATP binding, etc. (Fig. 5B) (Supplementary File 4).

We further confirmed the effect of the cytoplasmic backgrounds on gene expression by exploring a set of candidate genes (Supplementary File 5) which have been cloned and validated for their role in salinity tolerance in rice identified from a review of the literature. Visual inspection of heatmaps of the expression of these candidates reveal that the tolerant and sensitive plants from different cytoplasmic backgrounds have quite different expression patterns. Leaf samples under stress could be uniquely identified in the heatmap (Supplementary Fig. 4A) with a variable cytoplasmic effect. A similar pattern was observed for the root tissues (Supplementary Fig. 4B).

### Effect of treatment in tolerance and sensitive families

**Figure 7:**
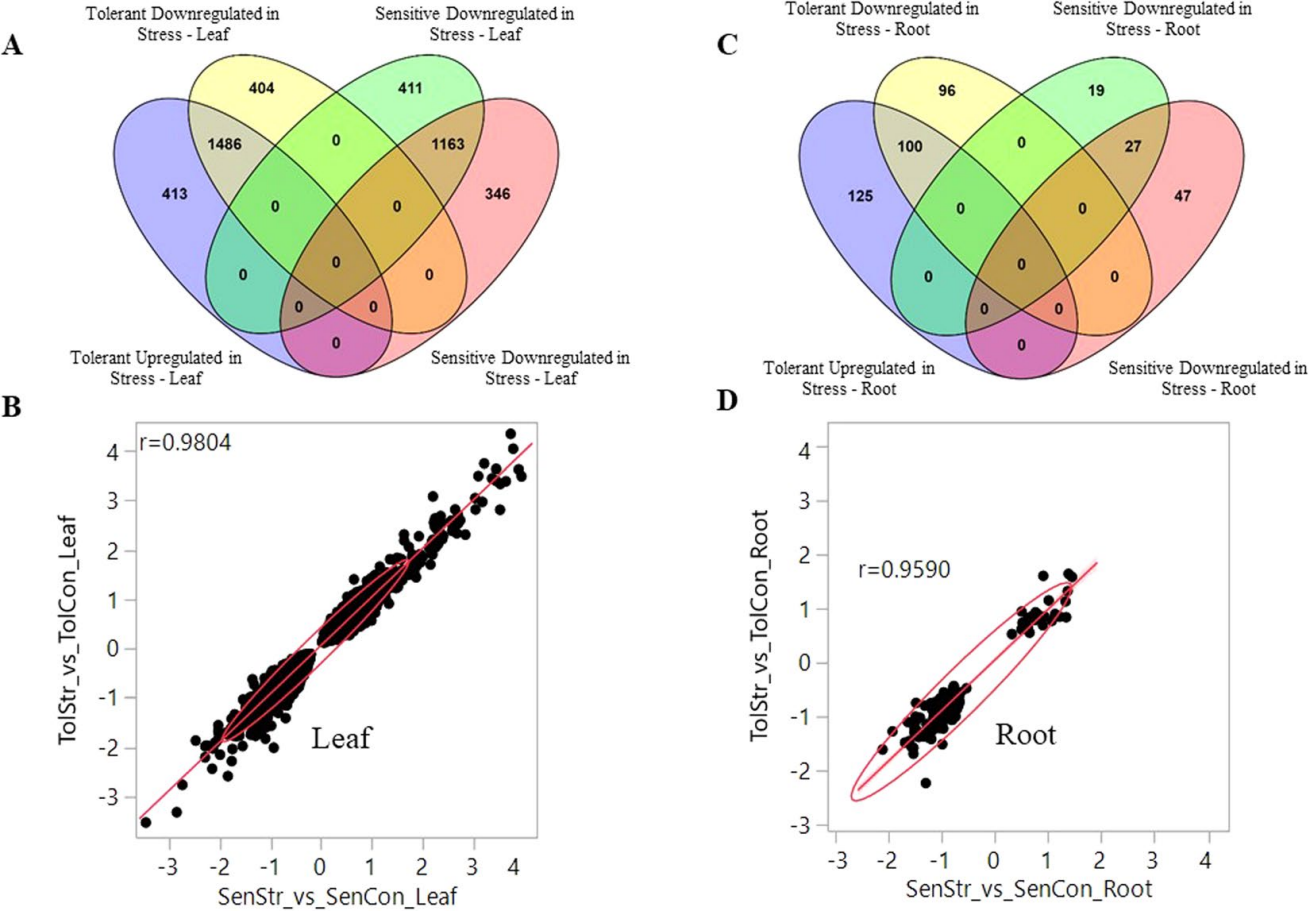
Similarities and differences of gene expression between stress group response under salinity stress. Differentially expressed genes were taken from tolerant stress vs sensitive stress contrast for both leaf and root tissues. Differential genes were separated by their direction and plotted to find similarities and differences. Fig. **A** Shows the overlapping and unique genes from this contrast in leaf tissue. These overlapping up and downregulated genes fold differences between sensitive and tolerant groups were plotted in fig. **B**. Fig. **C** Shows the number of unique and overlapping genes between sensitive and tolerant groups in root tissue. The overlapping genes fold differences were plotted in fig. **D**.

Research from several groups demonstrate that salt stress modulates the levels of expression a number of genes^6,36,44,45^. In this study, we identified genes whose expression may enable plants to adapt or tolerate salinity stress at the seedling stage. We observed that treatment had a strong effect on gene expression in leaf and root tissue (Table 1, Fig. 4). From the two-way interactions between stress group and treatment (stress group*treatment), we identified genes that were differentially expressed between sensitive stress vs sensitive control and tolerant stress vs tolerant control in leaf and root tissues. We detected overlapping genes between sensitive and tolerant plants in leaf tissue, the overlap was 64.5% for downregulated genes and 60.6% for upregulated ones (Fig. 7A). Notably, correlation coefficients between overlapping genes based on their fold change value for leaf was 0.98 (Fig. 7B).

In leaf tissue, there were 413 down regulated genes that were uniquely differentially expressed in sensitive plants and 404 genes for tolerant plants under stress. We used these unique DEGs for GO enrichment studies. We found 183 GO associations from downregulated genes that were unique to sensitive plants. These were enriched (p-value < 0.05) in different types of transporter activity including ion transport, ATP biosynthetic process, kinase activity, phosphorylation, phosphate- phosphorus metabolic process, gene expression, biological regulation, NADH dehydrogenase activity, photosynthesis, RNA splicing and regulation of cellular process. (Fig. 5C) (Supplementary File 6). On the other hand, there were 150 GO associations observed from the unique differentially expressed genes from tolerant plants under stress. Downregulated genes in tolerant progenies were enriched in sodium ion transport, potassium ion transport, intra-cellular protein transport, amino acid transport, cellular protein metabolic process, ribonucleoprotein complex. During salt stress, kinase activity and their phosphorylated genes play a crucial role to provide tolerance. In sensitive plants, there were 27 downregulated genes associated with kinase activity and 32 with phosphorylation. This indicates a reduction in crucial gene regulation under stress. At the same time, tolerant plants reduced their sodium and potassium ion transport during stress. In tolerant plants a downregulation of their protein translation process was also observed which can presumably reduce their energy expense during stress (Supplementary File 6).

There were 411 and 346 unique upregulated genes in sensitive and tolerant plants under stress, respectively (Fig. 7A). We found 96 GO association from these genes in sensitive plants and 121 GO associations from tolerant plants under stress. Upregulated genes unique to sensitive plants were enriched in cellular protein metabolic process, methionine metabolic process, oxidase activity, malate metabolic process, translational initiation-elongation, protein polymerization and oxidation reduction. On the other hand, in tolerant plants the upregulated genes were associated with different types of transport including ATP synthesis coupled transport, photosynthetic electron transport, ATP biosynthetic- metabolic process, trehalose biosynthetic process, and calcium ion binding. We also found that cellular protein metabolic process was downregulated in sensitive plants but upregulated in tolerant plants under stress. Moreover, upregulation in tolerant plants showed GO association with photosynthesis-related genes (Fig. 5C) (Supplementary File 6).

In root tissue, we also compared up- and downregulated genes from sensitive and tolerant progenies under stress. There were 31.2% downregulated and 29% upregulated gene that overlap between sensitive and tolerant progenies, respectively (Fig. 7C). Notably, correlation coefficients between overlapping genes based on their fold change value for root was 0.98 (Fig. 7D). As a result, we only analyzed the unique genes downregulated in sensitive and tolerant progenies. We found 121 genes uniquely downregulated in sensitive plants and 96 in tolerant plants under stress (Fig. 7C). The unique gene lists were also analyzed for their GO enrichment for both sensitive and tolerant plants. There were 52 GO associations enriched in sensitive plants and 83 in tolerant plant root tissue. Downregulated genes unique to sensitive plants were associated with various types of transport function including lipid transport, cation transport, metal ion transport, peptide transport, ion transport, electron carrier activity, chromatin assembly, DNA packaging, etc. (Fig. 5C). On the other hand, downregulated genes in tolerant plants included the trehalose biosynthesis process, different types of regulation activities including gene expression, kinase activity, phosphorylation, ATP biosynthetic process, etc. However, we observed only 18 genes uniquely upregulated in sensitive plants and 47 genes in tolerant plants which shows that tolerant plants under stress upregulate more genes than sensitive plants in root tissues. Unique upregulated genes in sensitive plants were associated (GO) with response to DNA dependent transcription regulation, metabolic process, and hydrolase activity. However, in tolerant plants enrichment was observed to be in protein ubiquitination, cell wall organization, signaling process, electron carrier activity. (Supplementary File 7).

### Effect of Time in tolerance and sensitive families

Time after stress was a major effect on differential gene expression in leaf and root tissue. We detected variable numbers of differential gene counts from leaf and root tissue (Table 1) at different time points with leaf tissue exhibiting fewer DEGs compared to root tissue across time and stress. We further inspected the set of genes that were differentially expressed between sensitive 24 hrs vs sensitive 72 hrs and tolerant 24 hrs vs tolerant 72 hrs in leaf and root tissues.

In leaf tissues, sensitive 24 hrs vs sensitive 72 hrs had 39 DEGs, including 21 genes that were downregulated and 18 that were upregulated (Table 1). Downregulated genes included a protease inhibitor, a leaf senescence related protein, a linker histone protein and ubiquitin fusion degradation protein. This set of 21 downregulated genes are associated with mRNA processing, cell wall modification, chromatin assembly and DNA conformation change based on their GO association studies. The upregulated list at 72 hrs in sensitive plants contained genes like a ras-related protein, a stem-specific protein, chloroplast 50 S ribosomal protein. These gene sets are GO associated with cytoplasmic part, ribonucleoprotein complex, cellular protein metabolic process, gene expression, endoplasmic reticulum membrane, signal transduction. (Supplementary File 8). On the other hand, we detected 23 genes from leaf tissue of tolerant plants that were differentially expressed between 24 hrs and 72 hrs. 13 genes were downregulated in 72 hrs and 10 genes were upregulated. Downregulated genes included a plastocyanin-like domain containing protein, leaf senescence related protein, no apical meristem protein, SNARE associated Golgi protein, F-box domain containing protein. These genes are GO associated with cellular biosynthetic process, ubiquitination, gene expression regulation. Upregulated genes in tolerant plants included bile acid sodium symporter family protein, zinc finger protein, glutathione S-transferase and transporter family protein. This list of upregulated genes in tolerant plants leaf tissues are GO associated with sodium ion transport, metal ion transport, integral to membrane function. (Supplementary File 8). In this set of analyses, it is clear that sensitive and tolerant plants had different types of expression variations over time even if treatment is not considered.

In root tissue, sensitive plants had about one-third fewer DEGs compared to their tolerant counterparts. Sensitive plants showed 32 downregulated genes and this list contained genes like peroxidase precursor, ABC transporter, zeaxanthin epoxidase, universal stress protein domain containing protein, etc. and these genes are GO associated with ubiquitination, gene expression, different types of metabolic process, unfolded protein binding and heat shock protein binding, etc. We detected 9 genes from sensitive plants that were upregulated in 72 hrs. These are zinc finger protein, kinesin motor domain containing protein, E2F family transcription factor protein. and these genes are GO associated with motor activity, transcription factor complex, translational elongation. In root tissues of tolerant plants, 99 genes were downregulated at 72 hrs and 43 genes were upregulated. Downregulated genes were peroxidase precursor, different types of kinases including calmodulin dependent protein kinase, peptide transporter, ubiquitination related protein, cell cycle control protein. These 99 genes were GO associated with kinase activity, ligase activity, peroxidase activity, response to oxidative stress. Some upregulated genes were sulfate transporter, hsp90 protein, calmodulin dependent protein kinases. These upregulated 43 genes are associated with transporter activity, gene expression, response to stress, cell differentiation, epigenetics. (Supplementary File 9).

### Gene expression variation between stress group under time of stress

In the linear model to detecting DEGs, we fit a three-way interaction between stress group, treatment and time point. We tested for genes that are variably expressed between sensitive and tolerant progenies in stress condition at different time points. To follow up with the genes list, we chose two contrasts group from the interaction. These two groups are tolerant 24hrs stress vs sensitive 24hrs stress and tolerant 72hrs stress vs sensitive 72hrs stress in both leaf and root tissues (Table 1).

In leaf tissue, we detected 53 genes differentially expressed between tolerant and sensitive plants at 24 hrs after exposure to salinity stress. In sensitive plants 30 genes were upregulated and 23 genes were upregulated in tolerant plants. We checked GO functional annotations of these DEGs. We observed different functional enrichments between tolerant and sensitive plants. In sensitive plants one gene annotated to abiotic stress stimuli (MYB family transcription factor, LOC_Os11g45740.1) was discovered, while in tolerant plants 2 genes were annotated in that same category of the upregulated genes list (PGR5 (LOC_Os08g45190.1) and OsRCI2-10 - Hydrophobic protein LTI6A (LOC_Os07g44180.1)). Interestingly, these two upregulated genes in tolerant plants under stress at 24 hrs were also upregulated in tolerant plants when we just studied DEGs between stress group without any interaction effects. Therefore, these two upregulated genes could be primed in tolerant plants for stress defense or adaptation to stressful condition. Three transporters were upregulated in sensitive plants, including two ABC transporters and an amino acid transporter. In the case of tolerant plants, one of the 2 upregulated transporters is a potassium channel transporter KAT1 (LOC_Os01g11250.1) often involved in salinity tolerance mechanism, while the other is undefined. We discovered a peroxidase precursor, two MYB family transcription factors, a WRKY1 transcription factors and a plastocyanin-like domain containing protein upregulated in sensitive plants. Two photosynthesis related genes, one zinc finger family protein, an ubiquinone oxidoreductase, an amine oxidase family protein and a heat stress transcription factor were also upregulated in tolerant plants (Fig. 5D) (Supplementary File 10).

In addition, we detected 28 DEGs from tolerant and sensitive plants after 72 hrs of stress. In sensitive plants 18 genes were upregulated and in tolerant plants 10 genes were upregulated. An Auxin-responsive SAUR gene family member (OsSAUR39) which responds to auxin dependent cell expansion is only upregulated in sensitive plants. There was also upregulation of a nonsense-mediated mRNA decay protein 3 (LOC_Os10g42320.1) in sensitive plants which functions to reduce errors in gene expression by eliminating mRNA transcripts containing premature stop codons. A DnaK family protein, UDP-N-acetylglucosamine-peptide N-acetylglucosaminyltransferase SPINDLY and a membrane associated transporter were also upregulated in sensitive plants. DnaK is GO annotated as stress responsive, cell differentiation and cellular morphogenesis related protein. UDP-N-acetylglucosamine–peptide N-acetylglucosaminyltransferase SPINDLY is GO annotated for signal transduction, endogenous stimulus responsive, transferase activity related protein. In tolerant plants a plus-3 domain containing protein was upregulated which is GO annotated as histone modification, transcription initiation, cell organization and biogenesis. Tolerant plants also upregulated acetyltransferase, heat shock protein, glutamyl-tRNA synthase and serine/threonine-protein kinase under stress at 72 hrs. Acetyltransferase is responsible for transferase activity, while heat shock protein is a stress responsive protein. Glutamyl-tRNA synthase shows catalytic activity associated with chlorophyll synthesis and serine/threonine protein shows kinase activity (Fig. 5D) (Supplementary File 10).

In root tissue, 4 DEGs were detected between tolerant and sensitive plants at 24 hrs of stress with each upregulated in tolerant plants. These genes include an integral membrane protein ((DUF6 containing protein (LOC_Os10g12500.1)), a blue-light photoreceptor PHR2 (LOC_Os03g22330.1), a glucan endo-1,3-beta-glucosidase precursor (LOC_Os02g04670.1) and ATOZI1 (LOC_Os11g06240.1). These genes are associated with DNA repair, responses to DNA damage stimuli, cellular response to stress, and primary metabolic process. At 72 hrs of stress, we detected 18 DEGs between tolerant and sensitive plants. Two of those 18 genes were upregulated in sensitive plants and 16 in tolerant plants. In sensitive plants, upregulated genes were associated with development, RNA binding, regulation of gene expression, epigenetics and other functions. One ras-related protein is upregulated in tolerant plants and is involved in transmitting signals within cells. There is one calmodulin dependent protein kinase (CaMK) which phosphorylates transcription factors and thus regulates the expression of responding genes (Fig. 5D). This CaMK is only activated when the concentration of intercellular calcium ions increases. GO annotations for the upregulated genes in tolerant plants were related to signal transduction, positive regulation of transferase activity, protein kinase activating G-protein coupled receptor signaling pathway, glucan metabolic process, carbohydrate-polysaccharide metabolic process. (Supplementary File 11). We observed that in leaf tissue photosynthesis related genes were only upregulated in tolerant plants which could be an adaptive strategy to ensure uninterrupted energy supplies to maintain cellular homeostasis during stress. In root tissue at both time points, tolerant plants exhibit more upregulated genes than sensitive plants. We saw signaling molecules like CaMK only upregulated in tolerant root which also could play some essential roles in upregulating essential genes to defend during stressful condition.

## Discussion

Well-known rice landraces like Pokkali and Nona Bokra are highly tolerant to salinity stress (damage scores or SES ~1 in a scale of 1–9) at the 2-week-old seedling stage. This stage is considered one of the developmental phases that is very sensitive to salt stress. However, progeny derived from these landraces are not as tolerant and do not show SES scores of less than 3 at the 2-week-old seedling stage^46^. One likely reason for this is that salt tolerance in these landraces is controlled by multiple genes likely interact in complex ways. Another cause is that donor rice landraces like Pokkali and Nona Bokra have a high vegetative biomass prior to panicle initiation and are significantly taller than the recipient commercial high-yielding genotypes. This phenology of the donor rice landraces results in favorable distribution and partitioning of the toxic Na^+^ in vegetative tissue and in particular in the older leaves, which is likely not possible in the smaller progenies which combine tolerance with high yield^47^. In addition, the heritability of the Na^+^ and K^+^ content, one of the main contributors towards seedling salt tolerance has been reported to be low^48^.

Horkuch belongs to the aromatic clade as opposed to Pokkali and Nona Bokra, both of which are grouped with the *indica* genotypes^16^. Moreover, upregulation of genes in Horkuch was found to have unique signature of its own in comparison to both Pokkali and Nona Bokra^6^. Therefore, we were interested in detecting the full complement of genes responsible for the tolerance of the rice landrace, Horkuch^6^, by analyzing a number of tolerant and sensitive progenies derived from its cross with a sensitive but commercial rice (IR29). By emphasizing the differences between replicated tolerant and sensitive individuals having similar average genetic backgrounds, we could also differentiate between the global response to salinity stress and specific expression in the two groups of progenies.

Leaf and root-specific differential expression was found to be distinct across all experimental conditions. Root specific DEGs involved transporter, kinase and electron carrier activity and in leaf, it involved differential upregulation of redox genes as well as photosynthesis related genes. Interestingly, roots seem to respond quickly and in general show stronger upregulation in gene expression by 24 hrs of stress compared to their control samples. In tolerant leaves on the other hand gene expression continues to be upregulated from 24 to 72 hrs. We hypothesize that roots detect salinity stress by 24 hours, signal appropriate responses to leaves, and then return to basal levels. A similar trend was observed for gene expression in tolerant Pokkali rice but not in sensitive IR29^45^. Tolerant leaves continue to respond up to 72 hrs. On the other hand, gene expression in leaves of sensitive progenies is higher at 24 hrs and decreases at 72, perhaps as a result of maladaptive gene expression responses in the sensitive roots.

Differential gene expression (DEGs) under control conditions in tolerant progeny leaves showed increased response in photosynthesis and redox-related genes and low expression of those concerned with apoptosis compared to sensitive leaves. Most interestingly, there was a significant over-expression of RCI2-10 (LTI6A) or rare-cold-inducible rice gene (Os07g44180) only in in tolerant leaves^49–51^. Moreover, the same gene was highly expressed only in tolerant progenies after 24 hours of salt treatment. Time sensitive early responses of this gene has been reported in salt tolerant rice where there was a relative lag of 8 hours in sensitive genotypes^50^. RCI2 proteins are a conserved family of low molecular weight, hydrophobic, membrane proteins involved in maintaining the integrity of the plasma membrane during cold, dehydration and salt stress conditions^50,51^. Genetic analysis showed that the *S. cereviseae* ortholog (PMP3) encoding a membrane protein, if mutated resulted in hyperpolarization of the plasma membrane potential, increased membrane permeability, and sensitivity of mutant cell lines to cytotoxic cations, such as Na^+^^49^. The rice genome has been shown so far to have a family of 11 RCI genes described as responsive to low temperature and salt^52^. Their function however remains unknown. In an earlier report with the same population at reproductive stages we reported expression of RCI2-10 in tolerant roots without salt, but higher expression in sensitive leaves after 72 hrs of salt stress^36^. At reproductive stage after 72 hrs, the RCI2-6 (LTI6B) gene was also differentially expressed in sensitive leaves. Such tissue-specific response has also been observed before with reports of developmental stage specific promoter elements^50^. It is tempting to speculate that once the plant is fully-grown and is preparing to initiate panicles, it needs to sense salt sooner, and so it is expressed in roots rather than in leaves. Upregulation of this gene in sensitive leaves maybe too slow to be effective.

Overall the GO enrichments in the tolerant group align with recovery strategies after having dealt with the effects of stress, while sensitive GO enrichments appear to align with persistent stress. The association of the tolerant progenies with differential upregulation of 2-peptidyl-prolyl cis-trans isomerase (Os05g01270.1, PPIase) in the absence of stress treatment could be an indicator of the primed response in these plants. This PPIase is functionally grouped as a cycophilin-like protein and stabilizes *cis-trans* transition during protein folding and has been shown to be implicated in conferring multiple types of abiotic stress tolerance^53–55^. In addition, the rice SCAMP1 or Secretory carrier-associated membrane protein 1 (Os07g37740.1) is also differentially upregulated in tolerant roots in the absence of salinity treatment. SCAMP1 is an integral membrane protein with four transmembrane domains (TMDs) localized to the PM and trans-Golgi network (TGN)^56^.

The salt stress treatment clearly induced the expression of genes related to calcium signaling, trehalose biosynthesis, photosynthesis as well as ATP synthesis in tolerant progenies. In contrast, sensitive genotypes upregulated genes involved in maintaining metabolic activity to cope with stress such as cellular proteins, methionine and malate^57,58^. At 24 hrs, the KAT1 gene encoding the inward rectifying K^+^ channel protein is activated only in tolerant leaves. This is likely a protective mechanism to reduce water loss and indirectly lower entry of salt water through transpiration^59^. There was also a differential upregulation of photosynthesis-related protein genes in tolerant progenies at 24 hrs of stress, e.g. Chlorophyll a/b-binding proteins. Higher expression of the latter have been known to confer abiotic stress tolerance, including salt stress in transgenic Arabidopsis plants^60^. At 24 hrs, on the other hand, sensitive progenies show up-regulation of a Myb transcription factor (Os11g45740.1) that may be involved in the jasmonate dependent (JA) defense response^61^. Continuous induction of the JA pathway may lead to excessive oxidative burst, leading to apoptosis and death^62^. Another 24 hrs upregulated WRKY transcription factor (Os01g14440) showed GO enrichment for negative regulation of transcription. In addition, a couple of ABC transporters were also upregulated in sensitive progenies at 24 hrs. One of these (Os01g52550) is an MDR or multi-drug resistant type. MDR-ABC transporters have been shown to be down-regulated under drought and submergence^63^. At 72 hrs of stress, the sensitive progenies show up-regulation of nonsense mediated decay (NMD), whereas, tolerant progenies are undergoing cell organization, biogenesis as well as chlorophyll synthesis in leaves. Such an adaptive early response was first reported in the well-known salt tolerant rice donor, Pokkali^45^.

At 24 hrs, roots in tolerant progenies have already responded to stress by upregulating metabolic repair activity and by 72 hrs are adjusting carbohydrate metabolism. On the other hand, sensitive progenies do not show any GO enrichment at 24 hrs and at 72hrs are attempting to re-activate ribosomal functionality. In the cross direction treatment interaction, progenies with IR29♀ showed higher activity in GO enrichment for cellular metabolic processes in leaf while the same was true for HK♀ progenies in root tissue. In the latter, however, in addition to cellular metabolism, there was also regulatory activity from phosphorylation and kinase enzymes as well as cation transmembrane activity. This may indicate immediate response in the HK♀ roots via protein tyrosine kinase activity and Ca^2+^ ions^64^. We are in the process of reporting QTLs associated with salt tolerance in the same population at both seedling and reproductive stages alongside and combining those with the RNAseq study here and reported earlier^36^ to identify the e-QTLs of importance responsible for the tolerance of the rice landrace, Horkuch.

## Conclusion

A picture of primed and early response to salinity stress emerges in tolerant seedlings as opposed to a slower response in sensitive ones. There is an activation of rare cold inducible genes (RCI2 family), regulatory kinases, photosystem genes, redox as well as, transporter genes to recover metabolic homeostasis in tolerant members of the population. Furthermore, once homeostasis is gained, activation of photosynthesis-related genes seems to lead the tolerant plants on the road to recovery. On the other hand, in sensitive progenies, the delay in activation of similar genes leads to upregulation of a lower number of redox genes, more apoptosis-related genes and a lack of response in photosynthesis-related genes. This lack of early activation likely prevents recovery of sensitive progenies from the effects of salt stress. Genetic mapping of the population is underway and expression QTLs (eQTLs) are being analyzed in order to strengthen our findings.

## Supplementary information

Supplementary Info

Supplementary File 1

Supplementary File 2

Supplementary File 3

Supplementary File 4

Supplementary File 5

Supplementary File 6

Supplementary File 7

Supplementary File 8

Supplementary File 9

Supplementary File 10

Supplementary File 11

## Data Availability

RNA-seq data analyzed here has been deposited in NCBI database under BioProject ID: PRJNA486511. SRA submission id: SRP158430 and can be accessed at https://trace.ncbi.nlm.nih.gov/Traces/study/?acc=SRP158430&go=go.
