# Supplementary material for: Gene Expression analysis associated with salt stress in a reciprocally crossed rice population
Source: Sci Rep. 2019 Jun 3;9:8249. doi: 10.1038/s41598-019-44757-4 (PMC6546764; doi:10.1038/s41598-019-44757-4)
Supplement: Supplementary file 1 — Supplementary Info [file 41598_2019_44757_MOESM1_ESM.pdf]

Supplementary File

Gene Expression analysis associated to salt stress in a reciprocally crossed rice population

Samsad Razzaque[1,2†], Sabrina M Elias[1,5†], Taslima Haque[1,2†], Sudip Biswas[1], G.M. Nurnabi Azad Jewel[1], Md. Sazzadur Rahman[3], Xiaoyu Weng[2], Abdelbagi M Ismail[4], Harkamal Walia[5], Thomas E. Juenger[2] and Zeba I Seraj[1*]

[1]Plant Biotechnology Lab, Department of Biochemistry and Molecular Biology, University of Dhaka, Dhaka-1000, Bangladesh
[2]Department of Integrative Biology and Institute for Cellular and Molecular Biology, University of Texas, Austin, Texas 78712, USA
[3]Plant Physiology Division, Bangladesh Rice Research Institute, Gazipur, Bangladesh
[4]International Rice Research Institute, Los Banos, Laguna, Philippines
[5]Department of Agronomy and Horticulture, University of Nebraska, Lincoln, Nebraska 68583, USA
[†]Contributed equally
*Corresponding author: zebai@du.ac.bd

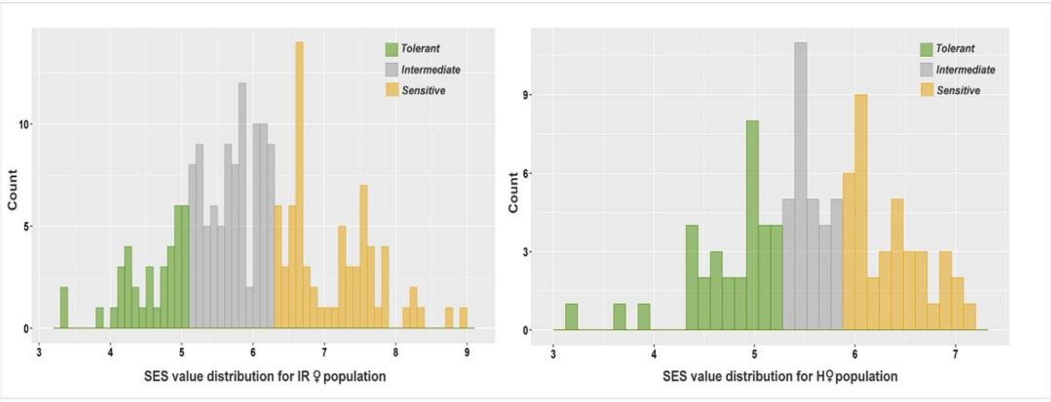

Fig 1: Distribution of SES score among the population

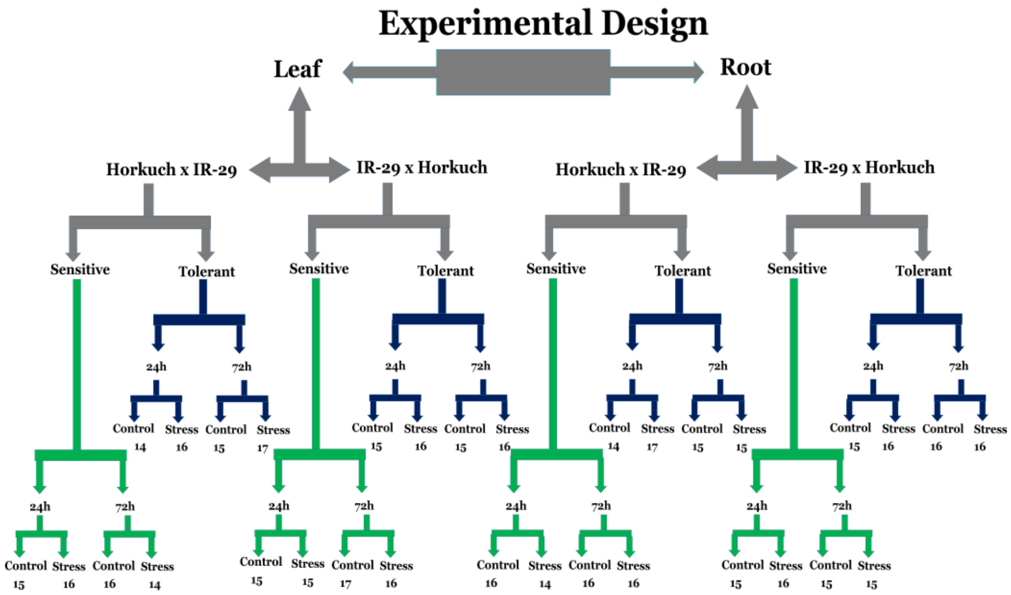

Supplementary File

Fig 2: The overall experimental design and the numbers of the samples used for RNAseq

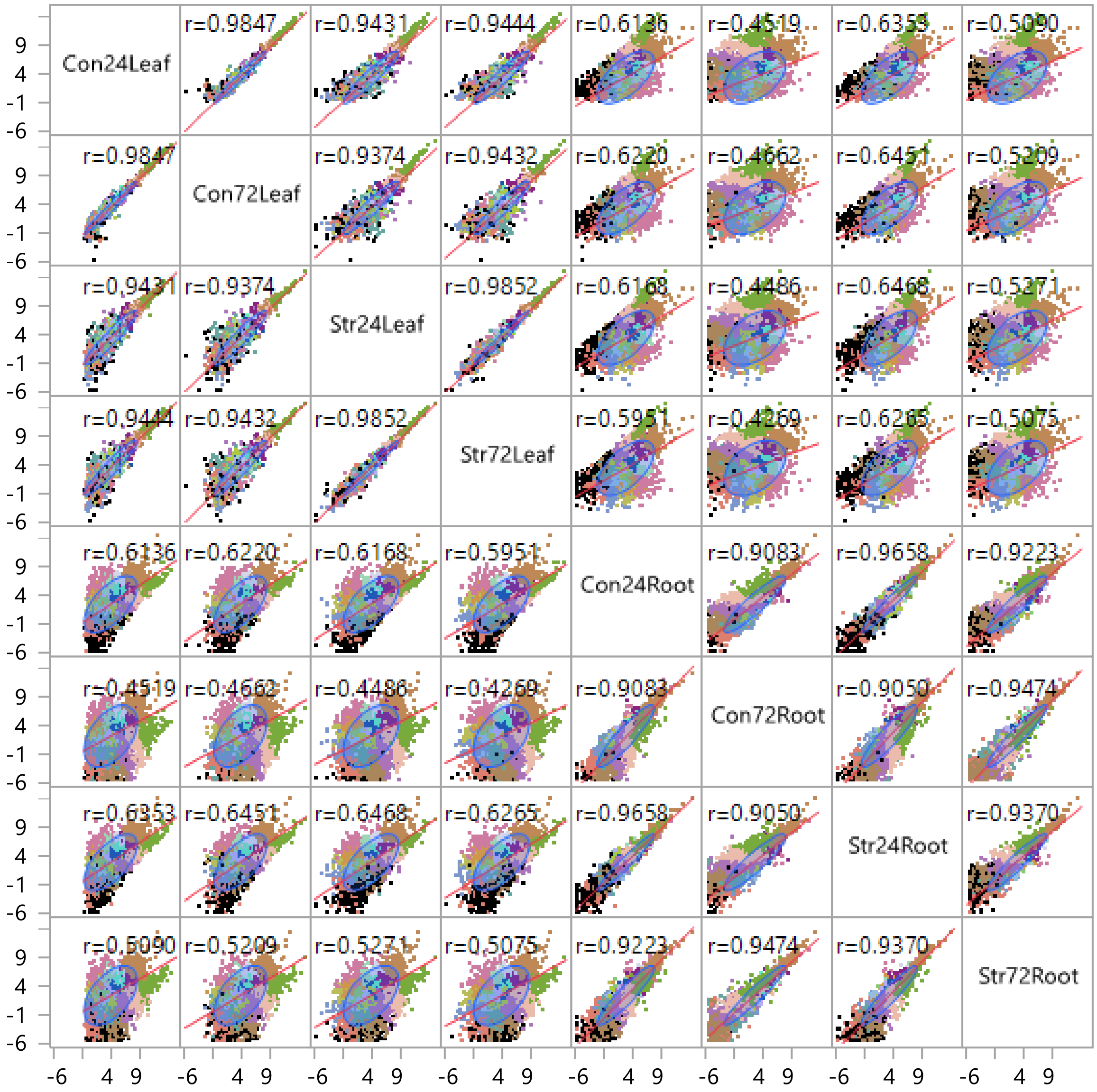

Fig 3: Bivariate scatterplots of expression counts in leaf and root tissue across various experimental factors. Global Pearson correlation coefficients (count data were log2 transformed) are presented for each pairwise comparison.

Supplementary File

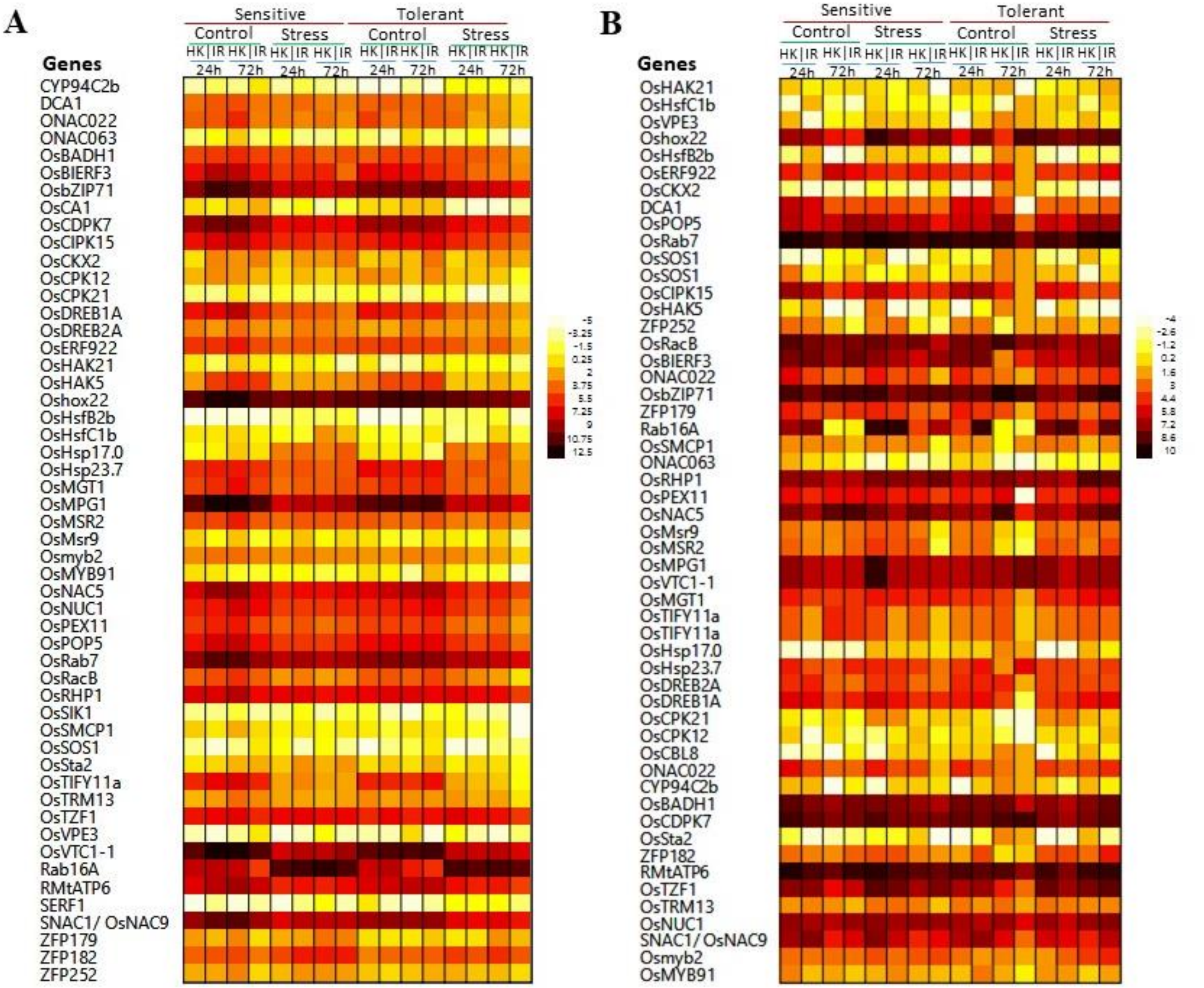

Fig 4: Heatmaps generated using genes that have been validated in earlier studies for their role in salinity tolerance in rice. Using this candidate gene set, we explored the expression change from our sensitive and tolerant groups by different cytoplasmic backgrounds plotted for both leaf and root tissue. At the Y axis, genes information has been provided for both the figs. Fig A shows the similarities and variability in leaf tissue and Fig B shows the same for root tissues.

Supplementary File

Supplementary File Legends

Supplementary File 1: Phenotypic data collected from the selected from different stress groups (tolerant and sensitive progenies).

Supplementary File 2: GO mapping of unique DEGs in sensitive and tolerant population. GO mapping of DEGs in leaf and root are shown in four separate worksheets of the supplementary excel file. Uniquely up and down-regulated genes and associated GOs are separately listed.

Supplementary File 3: Comparison of GO enrichments and metabolic pathways associated with unique up- and down-regulated DEGs in leaf with respect to cross-direction and treatment effects. Sheet 1 shows significant GO enrichments rom Horkuch cross direction and sheet 2 shows the GO classes from the IR29 cross directions.

Supplementary File 4: Comparison of GO enrichments and metabolic pathways associated with unique up- and down-regulated DEGs in root tissue with respect to cross-direction and treatment effects. Only Horkuch progenies showed significant GO enrichments which all are shown in this file.

Supplementary File 5: A collection of genes/ transcription factors that have been cloned and validated for their role salinity tolerance in rice and *Arabidopsis*. Leaf and root target genes are shown in two separate worksheets with the expression values in our study from different experimental factors.

Supplementary File 6: The effect of treatment over sensitive and tolerant progenies are shown by GO enrichments in leaf tissues. They are separated in 4 different sheets. Downregulated and upregulated genes associated GO classes are for both Tolerant and sensitive plants were grouped in different sheets.

Supplementary File 7: The effect of treatment over sensitive and tolerant progenies are shown by GO enrichments in root tissues. They are separated in 4 different sheets. Downregulated and upregulated genes associated GO classes are for both Tolerant and sensitive plants were grouped in different sheets.

Supplementary File 8: Differentially expressed genes from leaf tissues associated to time x phenotype effect have been shown with their fold change. Upregulated and downregulated genes for both tolerant and sensitive plants are presented in separated sheets.

Supplementary File

Supplementary File 9: Differentially expressed genes from root tissues associated to time x phenotype effect have been shown with their fold change. Upregulated and downregulated genes for both tolerant and sensitive plants are presented in separated sheets.

Supplementary File 10: The file shows the DEGs from leaf tissues under the composite effects of phenotype x time x treatment factors. Genes and their associated GO classes are shown here. Two sheets list the differentially expressed genes and their associated GO classes separated by time points.

Supplementary File 11: The file shows the DEGs from root tissues under the composite effects of phenotype x time x treatment factors. Genes and their associated GO classes are shown here. Two sheets list the differentially expressed genes and their associated GO classes separated by time points.
